# Cocaine-induced PRES in a previously normotensive patient: a case report and review of literature

**DOI:** 10.1186/s12245-026-01323-w

**Published:** 2026-07-31

**Authors:** Akshayaa Kumar Aggarawal, Aniruddh Shailesh Shringare, Mansoor Gazi

**Affiliations:** Internal Medicine, Walsall Manor Hospital, Birmingham, UK

**Keywords:** Seizure, Drug abuse, Emergency medicine, Vision loss, Stroke

## Abstract

**Background:**

Posterior reversible encephalopathy syndrome (PRES) is a clinicoradiological condition characterized by acute neurological manifestations and vasogenic cerebral oedema, typically affecting the parieto-occipital regions. Although acute hypertension is the most identified precipitating factor, the role of illicit substances such as cocaine is increasingly recognized yet remains underreported.

**Case presentation:**

We describe a case of a 30-year-old white female admitted with generalized tonic-clonic seizure associated with a hypertensive crisis (210/130 mmHg), followed by acute visual disturbance. Initial non-contrast computed tomography of the brain raised concern for bilateral occipital infarction; however, subsequent magnetic resonance imaging revealed bilateral symmetrical vasogenic oedema without diffusion restriction, consistent with a diagnosis of PRES. Urine toxicology screening was positive for cocaine metabolites. The patient achieved complete neurological and visual recovery following prompt blood pressure control and initiation of antiepileptic therapy. A follow up MRI showed near complete resolution of previously noted hyperintensities.

**Conclusion:**

This case underscores cocaine-induced PRES as an important differential diagnosis in young patients presenting with seizures, severe hypertension, and posterior circulation imaging abnormalities. Early recognition is crucial to prevent misdiagnosis as acute ischemic stroke and to avoid potentially irreversible neurological sequelae.

## Introduction

Posterior reversible encephalopathy syndrome (PRES) is a clinic-radiological disorder characterized by acute or subacute neurological symptoms, including seizures, altered mental status, headache, visual impairment, and focal neurological deficits, in association with reversible vasogenic cerebral oedema on neuroimaging. The parieto-occipital regions are most affected [1, 2]. Since its original description as reversible posterior leukoencephalopathy syndrome, the recognized clinical and radiological spectrum of PRES has broadened considerably, now encompassing atypical anatomical distributions, delayed onset, and severe or malignant forms of the condition [3, 4].

The underlying pathophysiology of PRES remains incompletely elucidated but is widely believed to involve disruption of cerebral autoregulation and endothelial dysfunction, leading to breakdown of the blood-brain barrier and subsequent vasogenic oedema [5]. Although acute hypertension is the most identified precipitating factor, up to 20% of patients may present without marked blood pressure elevation, underscoring the contribution of alternative mechanisms such as direct endothelial injury, inflammatory cytokine release, and toxic vascular effects [6]. As a result, PRES is increasingly recognized as a multifactorial syndrome rather than solely a manifestation of hypertensive encephalopathy.

Common precipitants of PRES include rapid fluctuations in blood pressure, pre-eclampsia/eclampsia, renal failure, cytotoxic therapies, and autoimmune disorders [7]. Autoimmune conditions associated with PRES include rheumatoid arthritis, systemic lupus erythematosus, Crohn’s disease, and scleroderma. Various toxins, including cocaine, lysergic acid diethylamide (LSD), and scorpion venom, have also been implicated [8]. There is substantial evidence that the use of chemotherapeutic and immunosuppressive therapies in malignancy increases the risk of PRES [9].Furthermore, in transplant recipients, immunosuppressive agents such as tacrolimus and cyclosporine are well-documented causes of PRES, leading to reversible white matter changes on neuroimaging. These changes typically improve following dose reduction or discontinuation of the offending agent [10, 11].

Cocaine is one of the important toxins implicated in the pathogenesis of PRES. As a potent sympathomimetic agent, it exerts significant central nervous system effects by inhibiting catecholamine reuptake, resulting in acute hypertension, cerebral vasoconstriction, and increased susceptibility to seizures [12, 13]. Beyond its hemodynamic effects, cocaine induces endothelial injury through oxidative stress, inflammatory mediator activation, and disruption of the blood-brain barrier-pathophysiological mechanisms that closely parallel those implicated in PRES. Although cocaine associated ischemic and haemorrhagic cerebrovascular events are well described, cocaine-induced PRES remains underrecognized and is frequently misdiagnosed, particularly when early non-contrast computed tomography (CT) findings resemble posterior circulation infarction [14].

PRES should be differentiated from other acute neurological conditions, including ischaemic stroke, vasculitis, encephalitis, and toxic leukoencephalopathy (TL), as management strategies and prognostic implications differ significantly. Diagnosis relies on clinical context and neuroimaging findings. MRI (Magnetic Resonance Imaging) in PRES typically demonstrates parieto-occipital vasogenic oedema on T2-weighted and FLAIR (Fluid-Attenuated Inversion Recovery) sequences, usually without significant diffusion restriction, in contrast to the cytotoxic oedema and restricted diffusion observed in acute ischaemic stroke [15]. Cocaine-induced toxic leukoencephalopathy is an important differential diagnosis, particularly in patients presenting with acute encephalopathy. Acute toxic leukoencephalopathy is characterised by variable neurological manifestations, ranging from mild cognitive impairment to severe neurological dysfunction, with MRI typically showing focal or symmetric restricted diffusion in the periventricular white matter on DWI (Diffusion-Weighted Imaging), with or without corresponding FLAIR abnormalities [16].

Visual disturbances, including transient cortical blindness, are prominent manifestations of PRES and reflect preferential involvement of the posterior cerebral regions. Several recent case reports have described cocaine-associated PRES presenting with seizures and reversible visual loss in young patients without preexisting hypertension, underscoring the importance of considering this diagnosis within the appropriate clinical context [17].

We describe a case of cocaine-induced PRES in a previously normotensive young woman who developed a hypertensive crisis, a generalized tonic-clonic seizure, and transient visual loss during an inpatient admission for mixed drug overdose. At the time of assessment, she had a reduced Glasgow Coma Scale (GCS) score [18], which improved by the following day.

This case underscores the diagnostic challenges arising from early CT findings suggestive of bilateral occipital infarction and highlights the crucial role of MRI in establishing the correct diagnosis. By presenting this report, we aim to add to the growing body of literature on cocaine-associated PRES and to reinforce the key clinical and radiological features that facilitate timely recognition and appropriate management.

## Case presentation

A 30-year-old white woman presented to the emergency department with the episode of abnormal body movement, followed by post-ictal confusion for nearly one hour. There was no associated tongue biting, urinary or faecal incontinence, or head trauma.

During the event, she was noted to be severely hypertensive, with a blood pressure of 210/130 mmHg, heart rate of 105 beats per minute, temperature of 38.4 °C, and oxygen saturation of 97% on room air. Neurological examination revealed a reduced GCS of 10/15 (E3, V2, M5), with equal and constricted pupils and symmetrical limb movements. No focal neurological deficits were identified. The seizure activity was successfully terminated with intravenous diazepam.

Her past medical history was significant only for an ovarian cyst. She had no prior history of hypertension, seizures, or other chronic medical conditions and was not taking any regular medications.

**Fig. 1:**
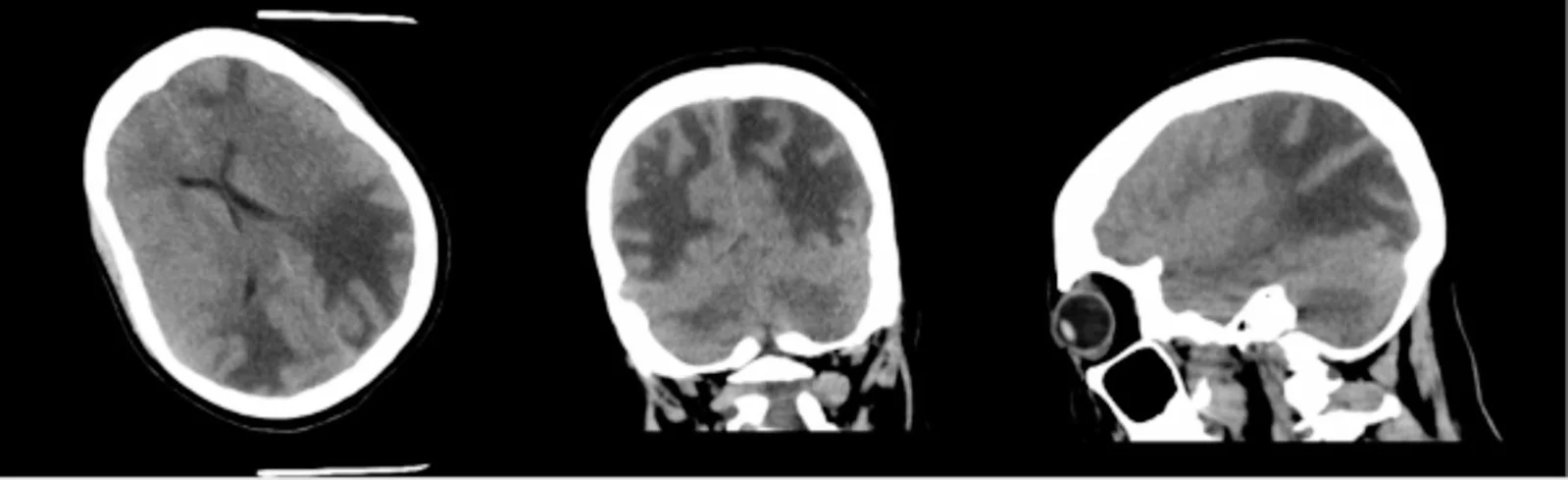
CT Head done after episode of seizures Non-contrast CT brain (axial, coronal, and sagittal reconstructions) performed following seizure activity demonstrates bilateral, symmetrical hypoattenuating areas involving the parieto-occipital cortices and subcortical white matter, with loss of grey–white matter differentiation consistent with vasogenic oedema

The non-contrast CT imaging of the brain demonstrated extensive bilateral low-attenuation changes within the occipital lobes, extending into the posterior parietal regions, with additional involvement of the left cerebellar hemisphere. These appearances were initially interpreted as bilateral posterior circulation infarctions. (Fig. 1)

**Fig. 2:**
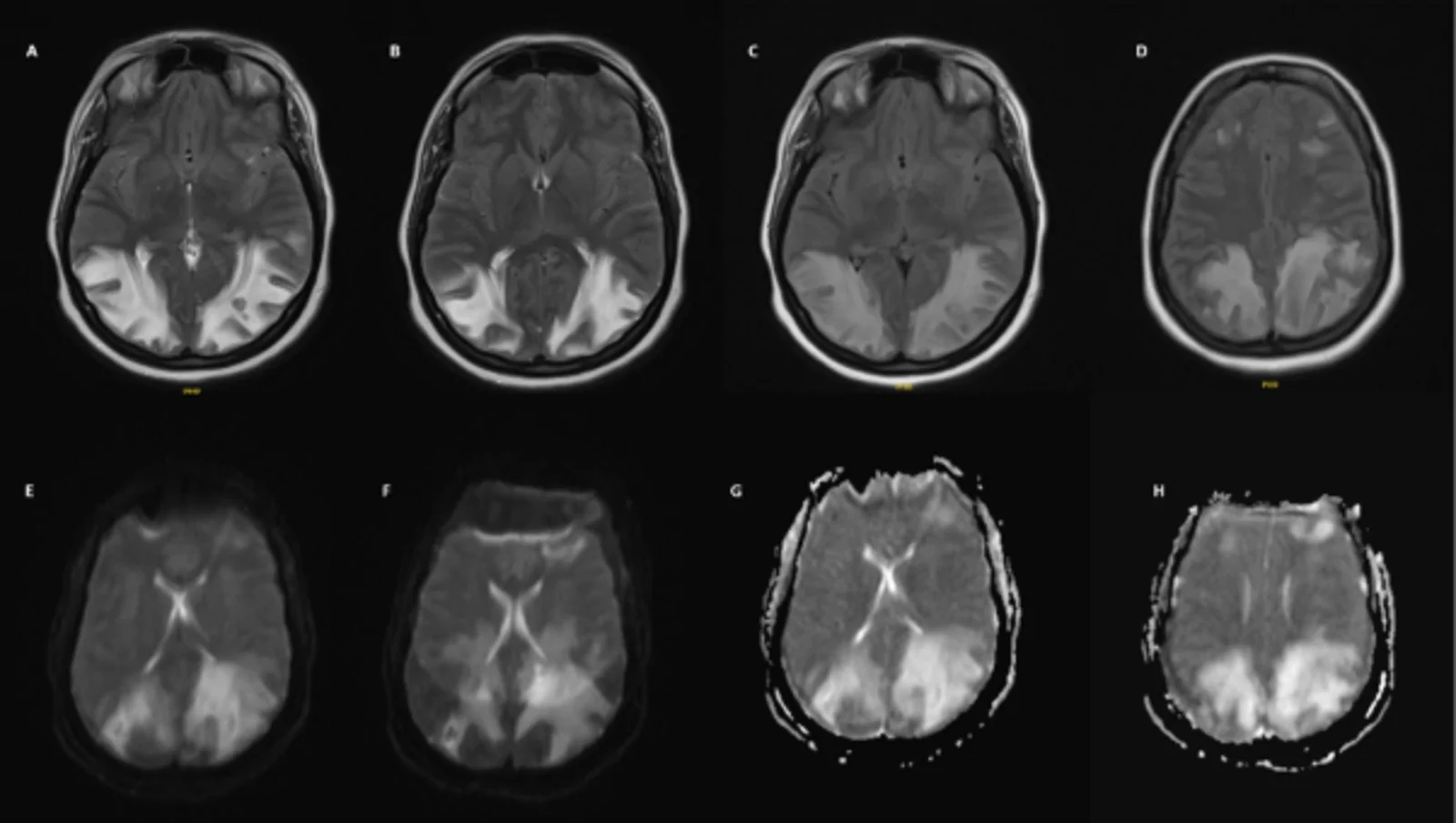
MRI scans of brain MRI demonstrating features of posterior reversible encephalopathy syndrome (PRES). (**A**–**D**) Axial T2- weighted and FLAIR images show bilateral symmetrical parieto-occipital hyperintensities. (**E**–**F**) DWI demonstrates corresponding signal without true diffusion restriction. (**G**–**H**) ADC maps show increased diffusivity, confirming vasogenic oedema. Findings are characteristic of PRES

Subsequent magnetic resonance imaging (MRI) of the brain revealed bilateral symmetrical hyperintense signal abnormalities on T2-weighted and fluid-attenuated inversion recovery (FLAIR) sequences involving the occipital, parietal, posterior temporal, and frontal subcortical white matter. Diffusion weighted imaging demonstrated no definite diffusion restriction, consistent with vasogenic rather than cytotoxic oedema. Collectively, these findings were highly suggestive of posterior reversible encephalopathy syndrome (PRES) (Fig. 2).

Urine toxicology screening was positive for benzoylecgonine, a metabolite of cocaine. The benzoylecgonine is the primary, pharmacologically inactive metabolite of cocaine, formed in the liver and excreted in urine (Table 1). On further questioning, the patient admitted to cocaine use disclosed a history of intermittent recreational use.

**Table 1:** Drug toxicology report

Test	Result Ref. Range (Units)
Morphine	Negative
Codeine	Negative
Dihydrocodeine (DHC)	Negative
6-MAM (Heroin)	Negative
Urine methadone	Negative
EDDP (Methadone Metabolite)	Negative
Buprenorphine	Negative
Norbuprenorphine	Negative
**Benzoylecgonine**	**POSITIVE**
Urine amphetamines	Negative
Methamphetamine	Negative
MDMA (Ecstasy)	Negative
Diazepam	Negative
Nordiazepam	Negative
Oxazepam	Negative
Temazepam	Negative
Lorazepam	Negative

Cocaine is converted into benzoylecgonine in the liver by carboxylesterases. It is the substance targeted in most urine drug tests for cocaine use

Despite no prior diagnosis of hypertension, she remained persistently hypertensive following the seizure episode and was commenced on antihypertensive therapy, with gradual achievement of blood pressure control. Antiepileptic treatment with levetiracetam was initiated.

Ophthalmological assessment two weeks later demonstrated complete resolution of visual symptoms, with visual acuity of 6/12 bilaterally and a normal fundoscopic examination. A follow-up CT scan performed at discharge showed persistent but stable bilateral occipital vasogenic oedema, without evidence of infarction or haemorrhage. The patient was discharged in a stable condition on antihypertensive and antiepileptic medications namely on amlodipine and levetiracetam tablets.

**Fig. 3:**
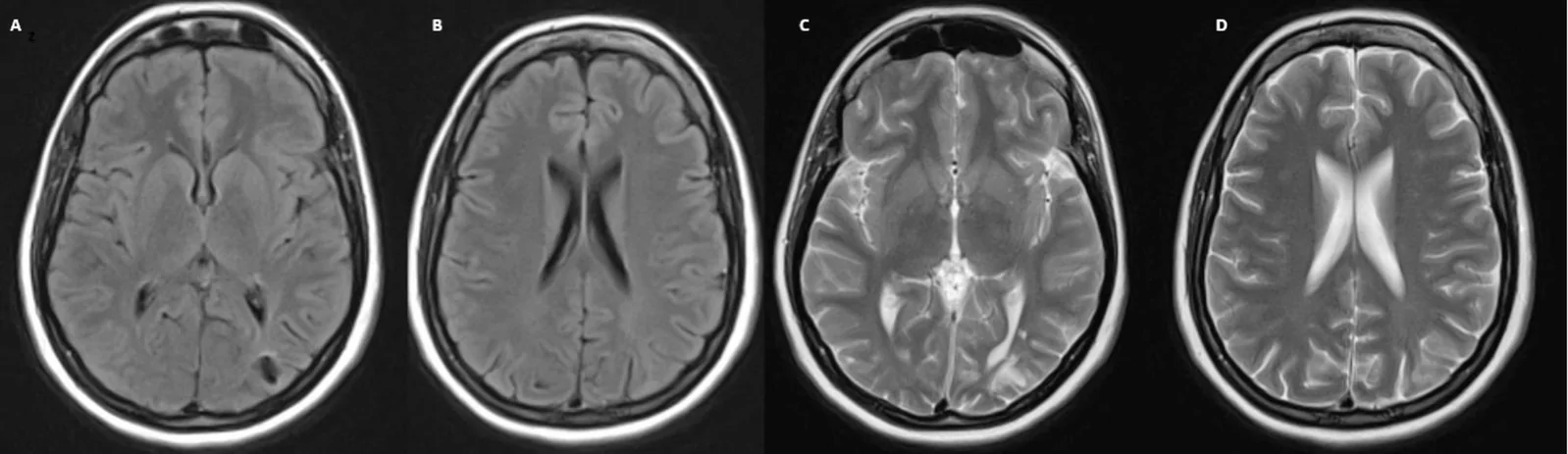
Follow-up MRI scans of the brain demonstrating resolution of posterior reversible encephalopathy syndrome (PRES). (**A**–**D**) Axial T2-weighted and FLAIR images show near-complete resolution of previously noted bilateral parieto-occipital hyperintensities, with restoration of normal cortical and subcortical signal

A repeat MRI scan was done in 4 months later showed near-complete resolution of previously noted bilateral parieto-occipital hyperintensities, with restoration of normal cortical and subcortical signal (Fig. 3).

Based on her clinical presentation, history of illicit drug use, positive cocaine toxicology, neurological findings, and characteristic CT and MRI changes, a diagnosis of PRES was confirmed.

## Discussion

Posterior reversible encephalopathy syndrome (PRES) is diagnosed on the basis of a compatible clinical presentation in conjunction with characteristic neuroimaging findings. The syndrome is defined by reversible subcortical vasogenic oedema and commonly presents with altered mental status, seizures, headache, nausea or vomiting, and visual disturbances, including visual field deficits or cortical blindness. Although imaging classically demonstrates predominant involvement of the parieto-occipital regions, more extensive or diffuse bilateral cerebral involvement has been described in a subset of cases [12].

### Pathophysiology of PRES

The PRES is a clinic-radiological entity characterized by failure of cerebral autoregulation and endothelial dysfunction, culminating in blood-brain barrier disruption and vasogenic cerebral oedema [1, 2]. While acute hypertension remains the most frequently identified precipitant, the syndrome is now understood to be multifactorial, with endothelial injury, inflammatory activation, and sympathetic overactivity contributing to its pathogenesis [3, 4].

PRES is associated with a range of precipitating factors and clinical settings, most commonly involving arterial hypertension and pre-eclampsia/eclampsia [7]. Additional risk factors include abrupt elevations in blood pressure, impaired renal function, autoimmune diseases, severe infections, transplantation, and exposure to cytotoxic or chemotherapeutic agents. PRES is frequently observed in patients undergoing allogeneic bone marrow or solid organ transplantation, particularly in the context of immunosuppressive therapy with agents such as ciclosporin and tacrolimus. It may also occur in the setting of septicemia and severe infections, often accompanied by shock and multiorgan dysfunction, as well as in autoimmune conditions such as systemic lupus erythematosus, scleroderma, and granulomatosis with polyangiitis [8].

One of the toxins that can cause PRES is cocaine. It exerts direct toxic effects on the vascular endothelium through oxidative stress, cytokine upregulation, and blood-brain barrier disruption-mechanisms increasingly implicated in PRES, even in the absence of persistent hypertension [4, 5]. Furthermore, cocaine being a potent sympathomimetic can provoke abrupt elevations in systemic blood pressure by inhibiting catecholamine reuptake, enhancing α-adrenergic stimulation, and inducing cerebral vasoconstriction [12, 13].

In this case, a previously normotensive young woman developed a hypertensive crisis temporally related to cocaine exposure, followed by seizures and visual impairment. The hypertension-hyper perfusion theory could explain our case. The theory proposes that a sudden surge in blood pressure overwhelms autoregulatory capacity, resulting in arteriolar dilation, cerebral hyper perfusion, blood-brain barrier breakdown, and vasogenic oedema. Conversely, the autoregulatory vasoconstriction hypothesis suggests that severe hypertension leads to cerebral vasoconstriction, hypoperfusion, and ischemia, with subsequent vascular endothelial growth factor-mediated increases in vascular permeability and oedema formation [17].

### Clinical features

The clinical manifestations of PRES vary depending on the underlying precipitating condition. In the setting of arterial hypertension, patients commonly present with headaches, whereas confusion is frequently observed in pre-eclampsia. In transplant recipients, including those undergoing allogeneic bone marrow or solid organ transplantation, PRES may present with nausea and vomiting. The use of immunosuppressive agents such as ciclosporin and tacrolimus is often associated with generalized seizures, which may progress to status epilepticus. In cases of septicemia and severe infections, particularly in the presence of shock and multiorgan dysfunction, cerebellar features may predominate. Additionally, in autoimmune diseases such as systemic lupus erythematosus, scleroderma, and granulomatosis with polyangiitis, visual disturbances—including cortical blindness, hemianopia, and blurred vision—may occur [8].

Visual symptoms occur in approximately one-third of patients with PRES and may range from mild blurring to complete cortical blindness [5]. Recent reports increasingly describe cocaine-associated PRES presenting with prominent visual manifestations, including reversible visual loss in young individuals without established vascular risk factors [17]. In our patient, severe but transient visual impairment resolved completely, with subsequent normal ophthalmological findings, underscoring the reversibility of PRES when promptly identified and treated. In contrast, delayed recognition or inadequate management-particularly of hypertension-may result in permanent visual deficits, intracerebral haemorrhage, or infarction [19].

Seizures are the next frequent presenting feature of PRES may both reflect and exacerbate underlying autoregulatory failure [3, 5]. Cocaine further lowers the seizure threshold through enhanced excitatory neurotransmission and cerebral vasoconstriction, increasing the risk of generalized tonic-clonic seizures [13]. In this case, seizure onset coincided with a marked hypertensive surge (210/130 mmHg), suggesting a bidirectional process in which seizure-induced sympathetic activation further amplified blood pressure elevation and vice-versa. Comparable seizure-associated hypertensive crises have been documented in cocaine-related and malignant PRES, highlighting the necessity of rapid and aggressive blood pressure control to prevent irreversible neurological injury [19, 20].

### Radiological investigations

Early non-contrast CT in PRES is often non-specific and may mimic ischaemic infarction, particularly when bilateral posterior hypodensities are observed [2, 3]. In this case, initial CT demonstrated extensive bilateral occipital and parietal low-attenuation changes that were initially interpreted as large posterior circulation infarcts. However, relative sparing of the calcarine cortex and paramedian occipital lobes-features more typical of PRES-helps distinguish this entity from bilateral posterior cerebral artery territory infarction [12].

This diagnostic challenge is well documented, especially in cocaine-associated cases, where clinicians may favour a vascular aetiology given cocaine’s established link to ischaemic and haemorrhagic stroke [21]. In contrast to PRES, true infarction usually conforms to a defined vascular territory and demonstrates diffusion restriction on MRI-findings not present in this patient.

MRI remains the diagnostic gold standard, characteristically showing symmetrical cortical and subcortical T2-weighted and FLAIR hyperintensities without true diffusion restriction, reflecting vasogenic rather than cytotoxic oedema [4, 5]. In this patient, MRI revealed bilateral symmetrical involvement of the occipital, parietal, posterior temporal, and frontal subcortical white matter, as well as the cerebellar hemispheres and brainstem, with preserved diffusion-features highly characteristic of PRES [2]. The presence of T2 shine-through without corresponding ADC (Apparent Diffusion Coefficient) restriction further excluded established infarction in our case.

### Differential diagnosis of PRES

PRES should be differentiated from other acute neurological conditions, including ischaemic stroke, vasculitis, encephalitis, TL, and toxic–metabolic encephalopathy, as management and prognostic implications differ significantly. The most frequent differential is primary or secondary headache, with approximately one-quarter of patients presenting with headache as the initial symptom in one study, followed by toxic–metabolic encephalopathy, likely reflecting the shared feature of confusion [22]. PRES also overlaps clinically with infectious and autoimmune encephalitis, particularly in presenting with seizures and altered mental status; however, encephalitis more commonly involves the hippocampus and cortical grey matter and may demonstrate haemorrhagic or inflammatory changes, whereas PRES typically shows reversible, white matter–predominant vasogenic oedema with relative cortical sparing [15].

TL represents an important differential diagnosis. It may result from cranial radiation, chemotherapy, environmental toxins, or recreational drug use, including levamisole-adulterated cocaine [18, 23]. The underlying mechanisms involve neuroinflammation, hypoxia–ischaemia, and direct axonal injury, producing diffuse white matter changes on T2/FLAIR MRI, often involving the centrum semiovale, corona radiata, and internal capsules. In contrast to PRES, TL typically demonstrates marked diffusion restriction on DWI, reflecting cytotoxic injury, and is often associated with slower or incomplete clinical recovery [13, 24]. Recognition of these distinguishing imaging and clinical features is essential for accurate diagnosis and appropriate management.

Cocaine-induced toxic leukoencephalopathy, a subtype of TL, is another differential diagnosis, particularly with the rise of levamisole-adulterated cocaine. Levamisole has immune-stimulating properties through monocyte chemotaxis and enhancing macrophage functions which could be responsible for possible immune-mediated neuronal toxicity. It might also potentiate the euphoric effects of cocaine by inhibiting dopamine reuptake and forming amphetamine-like metabolites [18, 23]. It typically shows confluent supratentorial white matter involvement with marked diffusion restriction and is often associated with slower or incomplete recovery compared with PRES [13, 23].

### Management

The cornerstone of management in PRES is prompt and careful control of blood pressure. Most published series report a favourable prognosis when PRES is recognized early and managed appropriately, with progressive blood pressure reduction resulting in complete resolution of clinical manifestations and radiologic abnormalities on magnetic resonance imaging (MRI) [12].

Acute management is primarily supportive and should include identification and correction of the underlying precipitating factor, most commonly uncontrolled hypertension. In patients presenting with acute severe hypertension, blood pressure should be reduced in a controlled and gradual manner, typically by no more than 20–25% within the first several hours, in order to minimize the risk of cerebral, coronary, or renal ischemia. A target mean arterial pressure of approximately 105–125 mmHg is generally recommended. Continuous intravenous antihypertensive infusions are often required to achieve stable and titratable blood pressure control [25].

## Conclusions

Posterior reversible encephalopathy syndrome (PRES) should be recognised as an important and potentially reversible neurological complication of cocaine use, even in young patients without pre-existing hypertension or conventional vascular risk factors. This case illustrates how cocaine-induced sympathetic overactivity and endothelial dysfunction can precipitate an acute hypertensive crisis, seizures, and transient visual loss, culminating in the characteristic radiological features of PRES. Early neuroimaging with computed tomography may be misleading and can mimic bilateral posterior circulation infarction, highlighting the pivotal role of magnetic resonance imaging in establishing an accurate diagnosis.

Prompt recognition of PRES and timely initiation of appropriate management-including aggressive blood pressure control, seizure treatment, and removal of the offending agent-are essential to prevent irreversible neurological injury. In the context of rising stimulant use, increased clinical awareness of cocaine associated PRES is crucial to avoid diagnostic error, inappropriate stroke-directed therapies, and adverse clinical outcomes. This case contributes to the growing body of evidence supporting the expanding aetiological spectrum of PRES and underscores the importance of integrating clinical presentation, toxicological findings, and advanced neuroimaging in the assessment of acute neurovascular syndromes.

## Data Availability

No datasets were generated or analysed during the current study.

## Abbreviations

PRES: Posterior reversible encephalopathy syndrome
CT: Computerised Tomography
MRI: Magnetic Resonance Imaging
FLAIR: Fluid-Attenuated Inversion Recovery
DWI: Diffusion-Weighted Imaging
ADC: Apparent Diffusion Coefficient
TL: Toxic leukoencephalopathy
GCS: Glasgow Coma Scale
LSD: Lysergic Acid Diethylamide
